# Methodological Considerations in Designing and Evaluating Animal-Assisted Interventions

**DOI:** 10.3390/ani3010127

**Published:** 2013-02-27

**Authors:** Cindy Stern, Anna Chur-Hansen

**Affiliations:** 1 The Joanna Briggs Institute, University of Adelaide, Adelaide, SA 5005, Australia; E-Mail: cindy.stern@adelaide.edu.au; 2 Discipline of Psychiatry, School of Medicine, University of Adelaide, Adelaide, SA 5005, Australia

**Keywords:** animal-assisted interventions, evidence based practice, qualitative, quantitative, methodology

## Abstract

**Simple Summary:**

There is a growing literature on the benefits of companion animals to human mental and physical health. Despite the literature base, these benefits are not well understood, because of flawed methodologies. This paper draws upon four systematic reviews, focusing exclusively on the use of canine-assisted interventions for older people residing in long-term care. Two guides are offered for researchers, one for qualitative research, one for quantitative studies, in order to improve the empirical basis of knowledge. Research in the area of the human-animal bond and the potential benefits that derive from it can be better promoted with the use of uniform and rigorous methodological approaches.

**Abstract:**

This paper presents a discussion of the literature on animal-assisted interventions and describes limitations surrounding current methodological quality. Benefits to human physical, psychological and social health cannot be empirically confirmed due to the methodological limitations of the existing body of research, and comparisons cannot validly be made across different studies. Without a solid research base animal-assisted interventions will not receive recognition and acceptance as a credible alternative health care treatment. The paper draws on the work of four systematic reviews conducted over April–May 2009, with no date restrictions, focusing exclusively on the use of canine-assisted interventions for older people residing in long-term care. The reviews revealed a lack of good quality studies. Although the literature base has grown in volume since its inception, it predominantly consists of anecdotal accounts and reports. Experimental studies undertaken are often flawed in aspects of design, conduct and reporting. There are few qualitative studies available leading to the inability to draw definitive conclusions. It is clear that due to the complexities associated with these interventions not all weaknesses can be eliminated. However, there are basic methodological weaknesses that can be addressed in future studies in the area. Checklists for quantitative and qualitative research designs to guide future research are offered to help address methodological rigour.

## 1. Introduction

It is widely accepted that animals can play a role in the physical and social health of some humans, although the mechanisms by which they do this remain uncertain. This is primarily due to the current lack of rigorous scientific research that has been conducted to substantiate the benefits of companion animals to human health. A number of authors have outlined the existing weaknesses in current research and the ways in which these weaknesses hamper the evidence for the role animals might play in human mental, physical and social health [1,2,3,4,5]. We explore the current literature base, discuss issues surrounding methodological quality, to suggest reasons for why these weaknesses continue to occur, and provide recommendations to ensure future research progresses and surpass current standards. This paper draws upon the findings of a series of four systematic reviews focused solely on older people residing in long-term care who received canine-assisted interventions (CAIs) [6,7,8,9]. This paper is not intended as a comprehensive critical review and synthesis of the relevant literature, but rather, is a critique of the methodological strengths and weaknesses of the existing knowledge base. The aim is to identify ways in which to strengthen the evidence base through rigorous methodologies.

### 1.1. Canines as Therapy

Animals have been used to improve the health and wellbeing of humans for many years [10]. The current term used to define this is animal-assisted interventions (AAIs), described as “any therapeutic process that intentionally includes or involves animals as part of the process or milieu” [11]. AAIs can be further classified as either animal-assisted activities (AAAs) (the utilization of animals that meet specific criteria to provide participants with opportunities for motivational, educational, and/or recreational benefits to enhance quality of life) [12] or animal-assisted therapy (ATT) (a goal-directed intervention directed and/or delivered by a health/human service professional with specialized expertise, and within the scope of practice of his/her profession) [13]. Canines are the most common animal employed because of their availability, trainability and consequently predictability and hence the terminology described above can be modified to canine-assisted interventions (CAIs), canine-assisted activities (CAAs) and canine-assisted therapy (CAT). 

Much research on CAIs, CAAs and CAT has focused on the use of canines with the elderly; specifically those living in long term care facilities [10]. Although use of animals as therapy in contexts such as residential aged care is becoming more common, little research has been conducted that examines the effects and experiences associated with their use. In the health sciences, the common practice used to establish whether an intervention has an effect on an outcome (*i.e.*, to prove causality) or to show at least an association between the intervention and the outcome involves the conduct of primary research in the form of experimental and observational studies. Performing this level of experimentation usually stems from anecdotal evidence and the undertaking of case reports and descriptive studies (*i.e.*, progressing from hypothesis generating studies to hypothesis testing studies). Ideally a systematic review that permits the pooling of individual high-quality studies and provides a summary statistic should be one of the final steps in establishing a solid scientific base to validate or refute each potential intervention/therapy. Although ideal, this progression does not always occur. 

Although animals have been utilized within long-term care facilities as well as the broader health care spectrum for many years [14,15], the published literature within this field has only emerged in the last 30 years. The current standing of research in this field is such that the literature base has continued to grow but it largely remains at the anecdotal, descriptive or case report level [2]. Culliton [16] wrote that much research in the field is colored by “strong sentiment” and data to prove any benefit were scarce. In 1984 Beck and Katcher [17] reviewed the available literature at that time and concluded that animals had either no impact or produced relatively small therapeutic gains. The amount of controlled experiments that have been undertaken over the last 30 years is limited and often hampered by methodological limitations and biases. Koivusilta and Ojanlatva [18] noted that not all scientific explorations have been founded on representative samples or statistically correct methodologies. Chur-Hansen, Stern and Winefield [1], in a discussion of the methodological challenges in drawing conclusions about the efficacy or otherwise of animal-assisted interventions, found that to date, the characteristics hampering studies three decades ago are still evident in current literature, a conclusion also made by Phelps *et al.* [10] in relation to elderly people specifically.

If the quantity of literature has continued to increase, why is it that the quality of this research has not continued to progress with it? Is this due to the field of inquiry being one that cannot be verified through scientific research due to its associated complexities, or is it simply that current standards are poor and need to be refined? In order to answer this question it is necessary to first explore the current literature base to describe some of the common variations in design, conduct and reporting.

The pool of knowledge in the area of AAIs originates largely from the USA, however a number of papers from Australia, UK, Japan and Europe have also been published. Health practitioners, in particular nurses, who have been involved in implementing some type of AAI and are recounting their experiences, have written many studies in the literature. The remaining papers come from academics and other researchers, and students undertaking postgraduate research. Papers are predominantly published in health-related and animal-related journals. 

### 1.2. The Systematic Reviews

The four systematic reviews on which this paper is based focused on the effectiveness [9], meaningfulness [8], appropriateness [6] and feasibility of CAIs [7] used in long term care settings. Eight studies were included in the effectiveness review with no statistical pooling possible. There was no restriction to the type of outcomes measured with the majority of studies focusing on emotional aspects as opposed to physical or social measures [9]. Two qualitative papers met the inclusion criteria for the review focusing on the experiences of being involved in CAIs. Limited meta-synthesis was possible and as with the first review, it demonstrated some short-term positive results [8]. The remaining two reviews [6,7] did not locate any papers meeting inclusion criteria for the reviews, even though they were both open to quantitative, qualitative and textual data. Of the literature that was available on these two topics, it was generalized and did not delineate between different age groups, settings or the animals used. Although the processes followed for this series of reviews was rigorous, the reviews were unable to solidly substantiate the assertions that animals improve health. 

The aim of searching for papers for a systematic review is to locate all available work (both published and unpublished) that relate to the review question and to then assess each paper to determine whether they meet the pre-specified inclusion criteria. The following descriptions are based on papers that were included in the reviews as well as those that did not meet the inclusion criteria but were reviewed in the process. 

## 2. Quantitative Research

### 2.1. Design and Conduct

The design and subsequent conduct of a research study is the pillar to undertaking a methodologically sound study. If time and resources permit, a pilot study is recommended as this can help avoid any potential issues that may arise and allows for modifications to be made to the design and procedure [2]. Ideally when attempting to determine the effect of an intervention on a certain population and a certain outcome, the gold standard is a randomized controlled trial (RCT). This infers that the selection of participants to either the intervention or the control group is purely by chance. While the RCT is considered to be the most rigorous study design it is difficult to randomly assign most species of animals to institutionalized individuals. Ensuring trials were truly random would require assigning residents to receive the animal intervention without some kind of screening for their feelings/fears towards the animal as well as their potential susceptibility towards allergies which would be unethical. Potential participants need to be screened and subsequently provide their consent. Interestingly, many studies do not report screening participants in this way, and nor is consent always reported.

If randomization occurred after this process (*i.e.*, following screening and consent) it could be at the facility level. Ideally a large number of facilities could be included in the study with the facility as the unit of randomization *i.e.*, each facility could be randomly assigned to either experimental or control groups [19,20]. Having the control group at a different facility to the intervention group potentially avoids the issue of controls knowing that the treatment is taking place, which could impact on their results [21]. 

If participants were not selected randomly and were self-selected (voluntary) it would be more likely that those people who had an interest in animals would want to be involved in the study compared to those who have never had an animal or had no interest in them. Of the studies located that stated they were randomized, many did not describe how the randomization process occurred [22,23,24,25,26].

Closely related to randomization is allocation concealment. Allocation concealment is another factor commonly not described in research studies. It was not clear in the majority of studies reviewed whether allocation to treatment groups was concealed from the allocator, since most did not clearly identify who the allocator was nor the method that was used [9]. Ensuring the sample is of a sufficient size is also important in designing a study, since one of the goals is to make inferences about a population from the sample. The sample should be large enough to produce sufficient power in order to undertake statistical analysis to detect an effect. Having a small sample size is one of the most common limitations noted in literature [19]. Sample sizes tended to average between 30 and 40: the largest sample size located was N = 80 [25]. Koivusilta and Ojanlatva [18] note that small samples make multivariate analyses impossible, although meta-analyses are an option, whereby results from individual studies are combined to increase statistical power. Within a chosen sample the outcomes of people who withdraw should also be described and included in any analysis. Again, this is not always done or reported. 

The ability to blind participants to treatment groups *i.e.*, so they would not know if they are receiving the active treatment or control is gold standard methodologically. In AAI research this factor is impossible to control for. Some researchers have advocated not advising participants of the study prior to the introduction of the animal to minimize the chances that this would influence their responses [27]. Blinding the investigator may be possible but is dependent on whether they are responsible for measuring outcomes and if these outcomes are reliant on observation at the time of the intervention. The deliverer of the intervention cannot be blinded however—they cannot be unaware that they are bringing an animal to humans for therapeutic purposes.

### 2.2. Population Characteristics

Although the majority of studies in this field have focused on specific populations such as older people, their characteristics are often extremely complex making it difficult to generalize results. To give this some perspective, a study undertaken by Marx *et al.* [28] that utilized a group of people with dementia had an average of 7.2 medical diagnoses and received an average of 9.5 medications. These factors would substantially impact on the ability to engage in the intervention thus making it difficult to find comparable groups. 

There are other factors that may impact on the ability to participate in the intervention including mobility, exercise and activity level, level of care required, cognition, hearing and vision levels, past history/experience with animals, attitudes towards and attachment with animals including the animal involved in the intervention, types of activities undertaken in the facility and staffing levels.

Cognition levels are frequently described in the literature; however the majority of the variables listed above are not. Past history/experience with animals [29,30] and medication usage [30,31] are two factors that were noted sporadically. The study by Kongable, Buckwalter and Stolley [29] was one of the few to mention physical problems of the population in the form of hearing impairment, physical restraint and chemical restraint in the context of impacting on interactions. Banks and Banks [23] also note hearing impairment as a potential confounder. Few studies have commented on the effects of AAI programs for people who dislike animals or on the risks associated with such programs [19]. It is crucial for details of possible confounders to be mentioned and accounted for in any study undertaken.

### 2.3. Intervention

Sellers [32] notes the disparity in language and foundational concepts used across studies in terms of the actual application of AAT. The use of words and phrases such as pets, companion animals, animals as therapy, and pet facilitated therapy are used as though they were interchangeable with the actual interventions provided often showing little comparability. The results of a quantitative review demonstrated that many papers classed the intervention as CAT however when it was described in the methods section all fitted under the definition used in the review for CAAs since interactions were unstructured [9].

One of the most notable disparities in the literature in regards to the intervention is the lack of consensus on the standards for administering interactions. Some canines remain leashed at all times while others are let off the lead to interact with participants. Some studies do not provide this information [24,25,26,33]. The level of interaction with the animal can include an individual simply watching the animal move and interact with others, to someone quite “hands-on” who is embracing the animal (patting, kissing, cuddling), or involved in grooming, walking or playing with the animal. It is often up to the discretion of the participant how little or how much they interact. In some cases the animal is owned by the researcher, members of staff or is part of an organization that undertakes AAIs. Coinciding with this is the influence of the researcher and handler (which in some cases is one and the same) on the interaction. 

Many papers are unclear in describing who was present during the interaction, with most stating that a handler, researcher and/or therapist were present. Often communication and interactions between participants and people is limited by the use of a predetermined script. Others play a substantial role in facilitating the interaction between the animal and the participant as well as generating dialogue between themselves and the participants. Hall and Malpus [34] suggest that human interaction may be responsible for facilitating any change and that the critical component of the intervention may be in fact the interaction with the handler and not the animal. 

In terms of the format or mode of delivery, interventions can be delivered individually or in a group environment and there could be one animal, multiple animals or a variety of species utilized. Wallace and Nadermann [35] advise that in the majority of cases animals are introduced to a large group of individuals, typically in the dayroom or lounge of the facility and that by utilizing this approach it may be difficult to determine if any of the beneficial effects observed are actually a function of the intervention *per se* or due to the generally elevated social activity level that exists in the room during the intervention. Conducting a session individually in one’s room may be a totally different experience to the group scenario detailed above. 

The breed of canine used in the intervention may impact outcome. Marx *et al.* [28] found that larger breeds compared to smaller breeds were more popular with participants. The size of the animal may be an issue if participants are wheelchair bound, if they have mobility problems or if they are concerned or intimated by larger animals. Some individuals may prefer one breed to another, which could impact on their experience. The age of the animal may also play a role—younger dogs/puppies may be more active than older dogs and participants may shy away from the more lively animals or vice versa. Lutwack-Bloom, Wijewickrama and Smith [21] recommend assessing dogs at baseline to ensure comparable behaviors, if multiple dogs are to be utilized. Most studies provide a description of the animal used, however it is rare for studies to compare one animal with another and to explain the reasoning behind selection of the animal.

As with administering the intervention there is no accepted standard in relation to the duration of each session or the frequency of sessions to provide to participants. There is an extreme variance in the duration of a session, which would obviously depend on the ability of the individual/individuals to interact and stay focused. For example Marx *et al.* [28] and Kramer, Friedmann and Bernstein [36] both utilized people with dementia as their participants of interest with the duration of each session potentially lasting for as little as 3 minutes. On the other hand some sessions have been noted as lasting for 90 minutes [37] while Kongable, Buckwalter and Stolley [29] and Walsh *et al.* [38] described sessions lasting 3 hours. If a facility housed a resident animal the duration of interaction could potentially be long. In terms of session frequency, visits are scheduled weekly, fortnightly or monthly with some facilities organizing multiple sessions per week [39]. Commonly though, sessions are weekly and like session duration, frequency would alter if the animal was a resident animal. Over the course of their study Kongable, Buckwalter and Stolley [29] changed from a visiting dog to a resident dog and suggested that because participants had previous interactions with the animal they may have been desensitized to the presence of the dog as a novel experience. As with medication interventions used to treat an illness, prescribing the correct dosage is vital with current AAI literature signifying this remains unknown.

Lastly the very nature of the intervention itself, *i.e.*, as an adjunct therapy, makes the ability to prove causation or even association difficult. Since the intervention of often provided in combination with an array of other interventions it is difficult to determine if the AAI alone is responsible for a change in outcome. 

### 2.4. Comparisons

The need for a control/comparison group is essential in ensuring that any change in outcome is attributable to the intervention and doesn’t simply occur naturally over time. For those studies that utilized a control group, some did not describe any details of what that actually entailed, rendering it worthless [21,40,41]. A handful of studies have used multiple treatment groups *i.e.*, one group for the intervention (animal and handler), one group for a control and another group for a comparison [24,25,42]. This scenario seems ideal as it considers the presence of the handler as a separate condition and assists in establishing if the interaction between the handler and human influences outcomes. It also allows for an alternative intervention to be tested. Some studies utilize a person/people as an alternative intervention [21,43].

If a study utilizes a controlled design, the control and treatment groups should be comparable at entry in terms of their characteristics and subsequently be treated identically other than for the named intervention. This is to ensure confidence in the results *i.e.*, any change in outcome could be attributable to the named intervention. This will be difficult to achieve due to both the complexities associated with the population and the differences between facilities (if utilizing multiple facilities) or even within a single facility. Lutwack-Bloom, Wijewickrama and Smith [21] acknowledge the potential for the Hawthorne Effect, whereby participants achieve better results due to the attention they receive in being part of the study or the novelty of the situation as opposed to the intervention itself.

### 2.5. Outcomes

The outcomes measured across current studies are highly variable; both in type and the way they are measured. Outcomes are either general behaviors or behaviors only measured during the interaction [20]. The lack of standardization of outcomes indicates the inability of statistical pooling and hence the overall unreliability of results. Phelps *et al.* [10] comment that often changes in behavior are limited to only one or a small number of the measured target behaviors potentially limiting the clinical utility of the changes. Whaley [44] suggested that the effects that animals have on social responsiveness might be deeper than that measured by eye contact or vocalization, which may explain the varying results. In other words the positive effects from touching an animal or the memories of past companion animals may be short-lived, lasting during and shortly after the interaction. Therefore studies using experimental controls, which tend to measure lasting results and studies asking for descriptive case reports of recounts of the session may produce different results [44]. Whaley [44] emphasizes that this does not make the effect less important and insignificant to the participant however ideally an intervention should aim to produce long-term results.

Many studies measure outcomes by observational means. Kongable, Buckwalter and Stolley [29] point out that data gathered by observation are vulnerable to distortion and experimental bias. In most situations the quality of data obtained is also threatened by the risk of human perceptual errors, such as the investigators’ interest and involvement with the study [21,29,34]. The influence of staff reactions to the animal may play a role in misinterpreting results such that it may motivate an increased frequency of interaction. Where possible a structured observational checklist should be developed and interrater reliability established. Videotaping has been recommended as the method of choice [29,45], since it allows continual review so things that were not obvious during the interaction may be examined later.

As well as measuring data by observation, studies tend to include outcomes that rely on self-reporting by participants (e.g., depression, mood, well-being). As mentioned previously this can prove challenging (e.g., residents could become confused) and lead to inaccurate reporting. As well as the participants, some studies rely on the subjective observations by staff, family or friends [44,46] and their expectations on the effects of animals on participants may bias their assessment [47]. Caution should be taken when interpreting these measures and where possible outcomes should be measured in a reliable way using standardized measures with validated scales/tools. Outcomes should be measured in the same way for all groups.

The studies in this area have overwhelmingly measured outcomes in the short term, commonly between 4 and 8 weeks. Few studies measure outcomes in the longer term; Lutwack-Bloom, Wijewickrama and Smith [21] followed up for 6 months; Barak *et al.* [48] followed up at one year while Crowley-Robinson, Fenwick and Blackshaw [49] had follow up at 23 months. It is important to establish whether changes in outcomes lead to any long term benefit and it is also important to determine if changes occur across different situations such as following the conclusion of the intervention when the animal is not present or on a day where the intervention is not being conducted [10]. 

### 2.6. Reporting

Many of the methodological considerations described above might have been addressed but were not reported in the available papers. For example not all studies mentioned that consent had been given to participate [22,24]. Williams and Jenkins [50] note that it is not always clear how ethical approval was sought to protect participants, particularly those with dementia who may have been unable to consent to the study.

It is also not always clear how the research is funded. There may be conflicts of interest with the research if it has been funded by bodies with vested interests such as the animal care industry [1]. A declaration of any conflict of interest should always be provided. 

Publication bias is a common occurrence in any type of research. Although many of the experimental studies did not produce statistically significant results, the authors tended to speak positively of the intervention and even go on to recommend it [9]. Although there may not have been any negative effects associated with the intervention it is hard to be sure since they were not mentioned. Research that finds no effects may not be published, and it is possible that research reporting negative findings may also be less likely to appear in published literature.

## 3. Qualitative Research

Qualitative studies are important in determining the experiences of people involved in AAIs. Although quite common in most areas of inquiry, there are more quantitative studies that exist in the field of AAIs than qualitative and therefore issues pertaining to quality and conduct can only be based on a small proportion of studies. The current evidence base lacks in-depth information from qualitative research conducted without prior assumptions [1]. Qualitative research has the advantage of being open-ended; themes may be identified that have not previously been considered as important and these may be pivotal in helping to understand the mechanisms at work in the relationship to health [1].

### 3.1. Design and Conduct

Generally qualitative research tends not to follow a standardized set of “strict” criteria like experimental research. There is a range of different methodologies that can be used to undertake a qualitative study, and within each one a variety of approaches/perspectives can be followed. Nevertheless, qualitative research must demonstrate trustworthiness and rigour, and adhere to strict guidelines in order to achieve these [51]. Qualitative approaches do not distance the researcher from the researched; researchers legitimately influence the analysis when they interpret the data [52]. The core to conducting a good quality study lies in its credibility (confidence in how well data and processes of analysis address the intended focus), transferability (the extent to which the findings can be transferred to other settings or groups) and dependability (seeks means for taking into account both factors of instability and factors of phenomenal or design induced changes) [53].

These aspects can be measured by (a) the congruity between the philosophical position adopted in the study and all aspects of its methodology, methods (research question, data collection, analysis) and interpretation, (b) the scale to which biases of the researcher are made explicit and (c) the relationship between what the participants are reported to have said and the conclusions drawn in the analysis [52]. There are limited studies available that address all of these factors or at least report on all of them making it difficult to determine how credible their results might be.

The researcher may influence the data with their beliefs and opinions; for example they could direct how and where the interview leads and as such it is important to describe the researcher’s stance (both culturally and theoretically) and the potential influence this could have on the research. 

The main approach to data collection is by interviews, usually structured to some degree and on a one-to-one basis. Interviews varied in length (anywhere between 15–50 minutes) and studies explored different perspectives in the form of residents and staff. It was not always clear if staff were interviewed because residents were too frail to participate (some were in Alzheimer’s special care units) [8].

Often the study was undertaken at a single facility and one interview was conducted. Winkler *et al.* [47] and Savishinsky [54] took a different approach and interviewed participants at multiple time points. Collecting data at different points of time would be useful to determine if feelings and experiences changed over time for example before, during, and immediately following the intervention and in the longer term. 

As with quantitative studies the sample sizes utilized are small (usually around 6–10 people), although unlike quantitative research, this in itself is not a limitation of a study. Limited background information about the participants was provided. It is important to know a person’s attitude towards animals, past experiences with them, and their cultural and religious values. As with quantitative research, aspects such as cognition, vision and hearing ability, medication usage and morbidities would impact on the participant’s experiences and subsequently on the ability to describe them.

Many studies were mixed methods studies and contained small portions of qualitative data, however since they were predominantly quantitative in nature this meant that limited qualitative analysis could be undertaken or if they were, were not reported [8].

Publication bias is also likely. It is unclear whether papers included all of their findings especially participant quotes. For example Kongable, Stolley and Buckwalter [55] did not clarify how many findings they actually had. Qualitative papers have the disadvantage of length: often only core themes or selected themes can be presented, meaning that information may be lost to the literature base.

## 4. Conclusions

Using the elderly and canines as foci, this paper has explored the current body of research available in the field of AAIs and has found that the majority of studies lack sound scientific methodology. The consequence of this is that the results of studies (both quantitative and qualitative) cannot currently confirm whether AAIs are therapeutically beneficial to human health.

To determine whether there is actual benefit (as opposed to current perceived benefit) more consistent research is required that follows sound process and methodology. Due to the many complexities associated with AAIs, the “perfect study” *per se* cannot be undertaken since some of the issues mentioned throughout this paper cannot be avoided (e.g., participant blinding and true randomization) however, knowing what methodological issues to address can help identify the failings and possible confounders [11]. Standardization of AAI methodologies is needed, where possible, so that meaningful comparisons can be made between studies [56]. Improved methodological approaches, even without standardization where this is unfeasible, will likely assist in the identification of mechanisms involved in the human-animal bond and the beneficial effects of the relationship [56]. Reviewing and synthesizing the literature has revealed that some currently limiting features can be minimized therefore the following checklists are aimed at those presently involved in or planning to undertake research in the area of AAIs. These are guides for researchers to consider and predominantly focus on aspects associated with study design and reporting. They are intended to supplement existing guides to reporting, such as CONSORT [57] and other existing checklists for quantitative and qualitative methods, but with explicit applicability to research related to the human-animal bond, and specifically AAI, AAA and AAT.

Some core methodological considerations for future quantitative research:

- Has a protocol been developed and appropriate approval sought?
- Is it possible to conduct a pilot study?
- Is randomization possible (at some level)?
- Is there an adequate sample size to demonstrate sufficient power?
- Has allocation to treatment groups been concealed from those responsible for assigning participants to intervention and control groups?
- Have participants consented?
- Has sufficient detail about the participants been provided?
- Are participant groups comparable?
- Have potential confounders (e.g., cognition, vision and hearing impairment) been accounted for and described?
- Have measures been taken to account for participants with limited ability to interact with the animal/s and researchers?
- Were there any withdrawals from the study and were they included in any analysis?
- Have aspects surrounding animal selection, duration, frequency, format, mode of delivery and sequence of the intervention been considered?
- Is there a control group that accounts for the presence of the handler?
- Have all treatment and control groups been adequately described?
- Is it possible to include another treatment group involving an alternative intervention?
- Will all treatment and control groups be treated equally other than for the named intervention?
- Is it possible to use multiple sites/facilities in the study?
- What outcomes will be measured and is it possible to use objective measures as opposed to self-reporting measures?
- Are outcomes measured using reliable and validated scales?
- If outcomes are to be measured via observation is it possible to videotape and follow a structured checklist?
- Will the outcomes be measured the same way across groups?
- Is it possible for those measuring outcomes to be blinded to treatment group?
- Has sufficient follow-up time been taken into consideration?
- Have all the above aspects been adequately described?
- Have the researchers acknowledged any potential conflicts of interest associated with the research?

Some core methodological considerations for future qualitative research:

- Has a protocol been developed and appropriate approval sought?
- Has the sample size been justified?
- What sampling method was used? Has it been clearly described?
- Is the philosophical perspective/stance behind the study acknowledged?
- Is the research methodology in line with the question/objectives of the study, methods for data collection, the representation and analysis of the data and interpretation of results?
- Have the potential influences of the researcher been considered and articulated?
- Has sufficient background to participants been provided e.g., attitudes towards animals or conditions affecting interaction?
- Is it possible to conduct data collection (e.g., interviews) at multiple points of time?
- Have details of the intervention (e.g., animal selection, duration, frequency, format, mode of delivery and sequence of the intervention) been considered?
- Have the participants’ voices been adequately represented?
- Are the numbers of findings/statements/themes/metaphors clearly stated?
- Have all the above aspects been adequately described?
- Have the researchers acknowledged any potential conflicts of interest associated with the research?
